# Polymer Processing and Performance: 2018–2019

**DOI:** 10.3390/polym12010220

**Published:** 2020-01-16

**Authors:** Francesco Paolo La Mantia

**Affiliations:** Department of Engineering, University of Palermo INSTM, Viale delle Scienze, Ed.6, 90128 Palermo, Italy; francescopaolo.lamantia@unipa.it

The section “Polymer Processing and Performance” of Polymers has published, in the last two years, 175 high quality papers investigating the processing and properties of several polymer based systems. In particular, about 110 papers have been devoted to different types of processing—from melt processing to laser technologies, to supercritical fluid processing, to 3D printing—considering many interesting polymer based systems, and, in particular, nanocomposites and nanobiocomposites. A relevant part of these papers discuss the 3D printing of different polymer systems. Several properties of the polymer nanocomposites—electrical, mechanical, viscoelastic, anti-UV and flame retardant—were investigated in 35 papers. Finally, various properties of polymers systems were discussed in the last 29 papers.

All of these papers represent not only well assessed important subjects, but also the new frontiers of “polymer processing”. In particular, an interesting review “Natural Polymers for Organ 3D Bioprinting” [1] dealt with the 3D printing of biodegradable polymer systems, and in particular, the printing of organs. This review aims to cover recent progress in natural polymers for bioartificial organ 3D bioprinting. “It is structured as introducing the important properties of 3D printable natural polymers, successful models of 3D tissue/organ construction, and typical technologies for bioartificial organ 3D bioprinting.”

Regarding the possibility of harnessing new biodegrade polymer systems for 3D printing for fused deposition, the authors in [2] showed that one possible candidate is the polybutyrate-adipate-terephthalate–polymer (PBAT) filled with wood flour. Both neat and composite PBAT filled with varying wood flour fillers were experimentally analyzed for 3D printing by extrusion from pellet forms. The results were positive and the addition of small quantities of the wood flour filler material was found to improve the thixotropic nature of the polymer composite and consequently the inter-strand and inter-layer coalescence.

Electrospray atomization was the subject of the paper “Supercritical Assisted Electrospray: An Improved Micronization Process” [3] where “A new process is proposed that can largely improve classical electrospray (ESPR) atomization, thanks to the addition of supercritical CO_2_(SC-CO_2_) to the liquid mixture, in which a polymer is dissolved, forming an expanded liquid. The consequent reduction of surface tension and viscosity allows the production of micrometric or nanometric particles of controlled size and distribution at a production rate up to one hundred times that of the traditional process. The new process was applied to particle generation from a very high molecular weight polyvinylpyrrolidone (PVP) and tested at different polymer percentages by weight and at different pressures.”

Multi-walled carbon nanotubes (CNT) can be considered as interesting candidates to develop wearable piezoresistive sensors with sufficient stretchability and high sensitivity because they exhibit outstanding sensitivities, a large stretchability, and excellent waterproof performance. In [4], “The printed fiber-shaped sensor demonstrated capabilities of detecting and differentiating human joint movements and monitoring balloon inflation. These results obtained demonstrate that the one-step printed fiber-like strain sensors have potential applications in wearable devices, soft robotics, and electronic skins”.

Among the papers that investigated more typical melt processing operations, the authors of “Lightweight High-Performance Polymer Composite for Automotive Applications“ [5], discuss “the possibility of applying microcellular injection molding process In order to reduce the cost of the produced parts, still preserving the main properties of the material”. In particular, the investigated material was “a glass-fiber-reinforced polyamide 66 (PA66), a material of great interest for the automotive industry because of its excellent properties, although being limited in application because of its relatively high cost. In particular, the influence of the main processing parameters on morphology and performance of PA66 + 30% glass-fiber foamed parts was investigated. An analysis of variance (ANOVA) was employed to identify the significant factors that influence the morphology of the molded parts. According to ANOVA results, in order to obtain homogeneous foamed parts with good mechanical properties, an injection temperature of 300 °C, a high gas injection pressure, and a large thickness of the parts should be adopted.”

Foam processing of a biodegradable polymer, Polylactide (PLA), was the interesting subject of the review [6]. “PLA has comparable mechanical properties to polystyrene (PS). However, the lack of melt strength is often referred to as a drawback for most foaming processes. One way to overcome this issue is the incorporation of chemical modifiers which can induce chain extension, branching, or cross-linking. As such, a wide variety of substances were studied in the literature. This work should give an overview of the most commonly used chemical modifiers and their effects on rheological, thermal, and foaming behavior. Therefore, this review article summarizes the research conducted on neat and chemically modified PLA foamed with the conventional foaming methods (i.e., batch foaming, foam extrusion, foam injection molding, and bead foaming).”

With regard to the performance of polymers, several properties and some peculiar behaviors have been published in these two years. Among them, the paper “Structure and Properties Study of PA6 Nanocomposites Flame Retarded by Aluminum Salt of Diisobutylphosphinic Acid and Different Organic Montmorillonites” [7] describes “two different types of organic montmorillonite, namely quaternary ammonium salt intercalated MMT (CMMT) and quaternary phosphonium salt intercalated MMT (PMMT), used as fillers in the flame-retardant polyamide (PA6) based on aluminum salts of diisobutylphosphinic acid (ABPA).” The introduction of OMMT improved the dispersion of the flame retardant particles independently of the type of OMMT. “From UL-94 and limited oxygen index (LOI) tests, PA6/ABPA/PMMT presented the best flame performance, with a UL-94 of V-0 and a LOI of 33%. In addition, the PA6/ABPA/PMMT presented the lowest peak heat release rate (pHRR) among the investigated samples. Combined with the char layer analysis, it can be deduced that the introduction of PMMT improved the dispersion of ABPA, and promoted the formation of more efficient network structure, before promoting more compact char structures, which finally resulted in improved flame retardancy.”

The oxidation resistance of polyphenylene sulfide composites based on montmorillonite modified by benzimidazolium salt was investigated in [8], where “Organic montmorillonite (MMT) modified by 1,3-dihexadecyl-3*H*-benzimidazolium bromide (Bz) was used to prepare polyphenylene sulfide (PPS)/MMT composites by melting intercalation. The PPS/MMT composites showed mixed morphology, being comprised of exfoliated and intercalated structures with slight agglomerates. The tensile property of PPS/MMT composites was significantly improved due to the good dispersion of the MMT nanolayers. The test results showed that the tensile strength retention of the PPS/MMT composites was higher than that of pure PPS after the oxidation treatment. Moreover, FTIR and XPS analyses were also used to evaluate the oxidation resistance of PPS composites. The FTIR analysis confirmed that adding MMT could better limit the damage of the C–S group and retard the generation of sulfuryl groups (–SO_2_–) during the oxidation treatment compared to pure PPS. The XPS analysis also suggested that the addition of MMT could reduce the chemical combination of the elements sulfur (S) and oxygen (O) during oxidation treatment. Furthermore, the MMT nanolayers could also promote the transfer of S from a C–S bond into an –SO_2_– group.

In situ prepared and characterized biobased poly(propylene 2,5-furan dicarboxylate) (PPF), or poly(trimethylene 2,5-furan dicarboxylate) (PTF)/clay nanocomposites were investigated in paper [9]. These two biobased polymers can be considered as candidates to replace fossil-based terephthalate (PPT) and naphthate (PPN). “A series of PPF based nanocomposites with the nanoclays Cloisite^®^-Na (MMT), Cloisite^®^-20A (MMT 20A), and halloysite nanotubes (HNT) were synthesized via the in situ transterification and polycondensation method. The effect of the nanoclays on the structure, thermal, and crystallization properties of PPF was studied with several methods”. “The insertion of the nanofillers in the polymer matrix altered the crystallization rates, and TGA results showed good thermal stability, since no significant mass loss occurred up to 300 °C. Finally, the degradation mechanism was studied in depth with pyrolysis-gas chromatography/mass spectroscopy, and it was found that β-scission is the dominant degradation mechanism”.

It is worth mentioning that about one half of the papers published in the section “Polymer Processing and Performance” in 2018 has been cited at least four times, and then more than the present value of the Impact factor of Polymers.
